# Partnerships to advance patient safety and address preventable harm: case studies from international health care leaders

**DOI:** 10.1093/haschl/qxag008

**Published:** 2026-01-14

**Authors:** Rachel Bonesteel, Robert Saunders, Tilithia McBride, Susan Sheridan, David W Bates, Mark McClellan, Charles H Kahn, Eyal Zimlichman

**Affiliations:** Duke-Robert J. Margolis, MD, Institute for Health Policy, Health Care Transformation, Washington, DC 20004, United States; Duke-Robert J. Margolis, MD, Institute for Health Policy, Health Care Transformation, Washington, DC 20004, United States; Federation of American Hospitals, Washington, DC 20001, United States; Future of Health, Washington, DC 20001, United States; Patients for Patient Safety US (PFPS US), United States; Division of General Medicine and Primary Care, Brigham and Women's Hospital, Boston, MA 02115, United States; Harvard Medical School, Boston, MA 02115, United States; Department of Health Policy and Management, Harvard School of Public Health, Boston, MA 02115, United States; Duke-Robert J. Margolis, MD, Institute for Health Policy, Washington, DC 20004, United States; Federation of American Hospitals, Washington, DC 20001, United States; Future of Health, Washington, DC 20001, United States; Department of Health Policy and Management, Tulane University School of Public Health and Tropical Medicine, New Orleans, LA 70112, United States; Future of Health, Washington, DC 20001, United States; Sheba Medical Center, Ramat Gan 52621, Israel

**Keywords:** patient safety, preventable harm, technology and digital health, patient and caregiver engagement

## Abstract

There have been notable improvements in patient safety in recent years; however, significant challenges remain in reducing the incidence of preventable patient harm. Supporting patient safety efforts is increasingly important given increasing complexity of care and changing health needs, especially with aging populations. Emerging technologies and capabilities open new possibilities to address longstanding patient safety problems. For example, predictive analytics to support provider decision-making, increased patient interest in engagement in their care, and artificial intelligence provide opportunities to further reduce harm. Many of these examples support a more proactive approach to patient safety by focusing on anticipating, predicting, and preventing patient harm; however, implementation is essential to avoid unintended consequences. Additionally, health care organizations oftentimes cannot accomplish this work on their own and strategic partnerships are crucial for continued improvement. This paper proposes a strategic focus for health care leaders as they build comprehensive plans to prevent harm from adverse events. Drawing on international case examples from the Future of Health Community, it outlines actionable approaches to partnerships that can be adapted and implemented across diverse health care organizations.

## Teaser

How can health care leaders implement comprehensive plans to prevent harm from adverse events and identify strategies to successfully scale patient safety initiatives? This paper outlines 3 key strategies from international health care leaders across diverse organizations.

Key Points

- International health care leaders continue to implement comprehensive plans to prevent harm from adverse events, including approaches that can be adapted and implemented across diverse health care organizations and key factors that support the successful scaling and spread of patient safety initiatives.
- Three key approaches include providing real-time measurement feedback through predictive analytics, engaging and empowering patients and families, and leveraging technology.

## Introduction

There have been notable improvements in patient safety in recent years; however, significant challenges remain in reducing the incidence of patient harm from unsafe care. Patient safety has become more important given the increasing complexity of care and changing health needs, especially with aging populations. Reports of harm remain high, with 1 in 4 patients experiencing harm in inpatient settings in the United States.^1^ Patient harm is also the 14th leading cause of the global disease burden.^2^ There is a clear need to undertake different approaches as patient safety continues to be an ongoing challenge, and true rates of patient harm are likely much higher than current self-reported data.

Emerging technologies and capabilities open new possibilities to address longstanding patient safety problems. For example, predictive analytics to support provider decision-making, increased patient interest in engagement in their care, and artificial intelligence (AI) provide opportunities to further reduce harm. Many of these examples support a more proactive approach to patient safety by focusing on anticipating, predicting, and preventing patient harm; however, implementation is essential to avoid unintended consequences.^3^ Additionally, health care organizations oftentimes cannot accomplish this work on their own and strategic partnerships are crucial for continued improvement. Patients, families, and patient organizations provide important perspectives for identifying sources of harm, co-developing patient safety solutions, and supporting shared decision-making and communications that reduce harm.

While new tools and strategies offer fresh momentum, there remains ongoing debate about how to set effective and accountable goals for improving patient safety. Some frameworks advocate for the elimination of all preventable harm, while others call for measurable reductions in harm over time. Central to both perspectives is the shared commitment to doing everything possible to protect patients and continuously improve care.^4^ Studies suggest that a significant portion of harm can be prevented through robust safety practices, better communication, and system-level improvements.^5^ At the same time, much of the existing evidence base for patient safety continues to focus on the acute care setting, despite the growing shift of health care delivery to outpatient, home-based, and community environments.

This paper proposes a strategic focus for health care leaders as they build comprehensive plans to prevent harm from adverse events. Drawing on international examples, it outlines actionable approaches that can be adapted and implemented across diverse health care organizations. The paper also identifies key factors that support the successful scaling and spread of patient safety initiatives.

## Approach to identifying global evidence to advance patient safety efforts

Established in 2018 by Sheba Medical Center, and formally incorporated in 2022 headquartered in Washington, D.C., Future of Health (FOH) is an international community of over 50 senior health leaders from across the globe.^6^ Membership includes hospital executives, policy makers, academics, payers, and senior-level health sector leaders.

In partnership with the Duke-Margolis Institute for Health Policy, the research team reviewed peer-reviewed and gray literature and engaged external experts including academic scholars, health care providers, health delivery organization executives, patients, and commercial vendors. FOH members participated in virtual meetings, case study interviews, and an in-person summit in Cape Town, South Africa in November 2024. At the summit, members reached consensus on key focus areas to advance patient safety through group discussions and facilitated voting sessions. Furthermore, FOH members shared case examples of innovation, highlighted experiences and considerations in implementing different initiatives, and identified best practices across international health systems. Where appropriate case examples were identified, the research team has followed up with individual open interviews for a deeper dive. Through this methodology, we have identified 3 key focus areas that are illustrated through case studies that present evidence in action (Table 1).

**Table 1.** Evidence in action across key strategies to advance patient safety.

Patient safety strategies	Evidence in action: international case examples
Providing Real-Time Measurement Feedback Through Predictive Analytics	Health systems in the United States and Australia have partnered with Pascal Metrics, a Patient Safety Organization, to leverage Electronic Health Records (EHRs) and predictive analytics technology to identify preventable adverse events and respond in real-time.
Engaging and Empowering Patients and Families	SingHealth has championed patient and caregiver engagement by partnering with the SingHealth Patient Advocacy Network (SPAN). SPAN involved patients in quality improvement, process design, patient experience, and facility design projects, among others to implement initiatives to advance patient safety and reduce preventable adverse events through patient engagement efforts.
Leveraging Technology	Sheba Medical Center has implemented a medication safety initiative to address preventable medication-related errors. Sheba partnered with MedAware, an AI-enabled medication safety monitoring platform to support intervention and improvement efforts.

## Reducing error through real-time safety measurement feedback

An important strategy for health care safety is providing real-time feedback to clinicians and health care professionals on potential harms, which can both prevent harm and help organizations understand where care needs to be redesigned. Measuring patient safety has traditionally been reactive in nature, relying on organizations to voluntarily report incidents of harm after they have already occurred.^7^ Voluntary reports undercount the true rates of harm, and are often significantly delayed from the event. Increasing availability of electronic health record (EHR) data can allow for real-time measurement, with future goals to identify risk and prevent harm from occurring.

### Evidence in action

Pascal Metrics is a Patient Safety Organization (PSO) that provides a learning environment to collect and analyze patient safety data and provide feedback to clinical teams and patients.^8^ The platform leverages routine electronic measurement of patient data to monitor care in real time, utilizing existing EHR standards such as the international Health Level 7's Fast Healthcare Interoperability Resources.^9^ By using the EHR directly, it can identify a range of adverse events in close to real-time, and also identify risks that can lead to future harm. Given the challenges of EHR alert burden, the software leverages AI to minimize false positives. The program has been implemented in a number of hospitals and health systems across the United States and Australia and has been found to achieve over 25% or more all-cause harm reduction and over 60% of specific harm reduction across client systems, according to Pascal Metrics internal Community Collaborative data.^10^

Pascal Metrics has been utilized in various studies aimed at detecting and reducing harm. In one study, several health systems partnered with Pascal Metrics to evaluate the use of an automated all-cause harm trigger system to detect and address harm. Results found one hospital reported a significantly higher number of adverse events detected using this method (2696) compared to those identified using the standard voluntary incident reporting method (132).^11^ By using this approach, inpatient facility all-cause harm was reduced by 71% and specific harm reduction was reported as well (eg, 63% reduction in harm from preventable hypoglycemia). The study also found that transitioning to a real-time review process improved efficiency compared to the manual retrospective review, reducing the time needed for record review. In this case, manual review took approximately 20 minutes per record and a nurse reviewer could review 20 records in six and a half hours.^12^ In contrast, automated triggers allow a nurse to review the same number of records in 1 and a half hours.^11^ The automated approach can identify up to 10 times the level of serious harms compared to traditional voluntary methods.^10^

### Implementation considerations

Health system leaders emphasize that measurement often needs to be accompanied by clear communication about the goals and vision for patient safety, including preventable harm reduction. Communications can be reinforced by dashboards and feedback showing measured progress toward the vision and goals along with complementary incentives and support. Furthermore, leaders will need to measure and take steps toward advancing a positive safety culture, which is necessary for long-lasting change. Barriers to adoption include an entrenched safety infrastructure based around incident reporting systems, concerns about legal and reputational risk from the detection of many safety events, and challenges in how to respond to safety problems with limited clinical resources.^7^

Another implementation challenge for providing real-time feedback is concern over alert fatigue, as there have been many tools that provide alerts for potential safety issues, and the clinical team can become overwhelmed (and ignore) alerts that do not appear relevant. Pascal Metrics has implemented strategies that have been leveraged to reduce false alerts, including utilization of AI tools to identify only meaningful alerts. A similar implementation challenge is improving clinical workforce workflows, given the level of burnout and shortages worldwide. Pascal Metrics has worked with health care organizations on how to share data. Instead of sharing safety data with all front-line clinicians, who may not have time to review and act on it, they have found that it can be more useful to share with other groups in the organization (eg, shared at the unit management level, among quality safety teams, or at the regional or system level).

Another important consideration is how to receive data feedback without liability concerns. As a PSO, Pascal Metrics has special legal protections so that it can share patient safety information. As health systems across the globe consider investment in real-time harm reduction technology to support provider intervention, leaders can consider the value of joining a PSO like Pascal Metrics, which provides access to a data repository and allows hospitals to share patient safety and adverse events data in a secure environment free from liability concerns.

Another implementation issue, highlighted in more detail in a later section, is how to engage patients in patient safety initiatives. For example, Pascal Metrics participated in a clinical trial that explored the impact of providing patients, caregivers, and families with access to an individual data dashboard to engage in their care by understanding the risks for harm, which allowed them to monitor and intervene to prevent harm. This dashboard was available as a mobile app, in addition to desktops and laptops for both patients and their caregivers. Results from the trial found lower 30-day readmissions and 30-day mortality, in addition to secondary outcomes including strong patient acceptance and value and no increase in fear response to being privy to the information.^13^

## Engaging and empowering patients and families in improving patient safety

Across the globe, patients and families increasingly expect to be active and involved participants in their care journey, driven by a desire for greater autonomy, transparency, and personalized treatment. Furthermore, patients are experts in their health situation, symptoms, and bodies. There are examples where patients and caregivers have been effective partners in contributing to improved safety during their care journey, in reporting harm, in advocacy and awareness raising, and in the co-development of patient safety solutions.^14^ For example, during the patient care journey, self-directed patient safety approaches are gaining momentum, where patients and families use digital tools to actively manage their health and prevent harm.^15^ Another example of patient empowerment and engagement at the direct care level is the use of the Pascal Metrics mobile app.

At the organizational level, health systems across the world have introduced opportunities for patients, families, and caregivers to give feedback on their care engagement plans and other operational considerations that can identify potential or actual harm. For example, the operationalization of high-functioning Patient and Family Advisory Councils (PFACs) across health care systems in New York State in the United States reported lower rates of pressure ulcers, sepsis, septic shock, 30-day readmissions, and better patient satisfaction scores when compared with hospitals with low-functioning PFACs.^16^

### Evidence in action

Patients, families, and caregivers have participated in organizational-level efforts to improve care and patient safety through PFACs and boards across the world, including in North America, Europe, and Australia, and, in recent years, in Asia. As an illustrative example, an established PFAC in a US health system with an initiative focused on sepsis improvement worked with its family advisor to establish a triage screening system in the emergency department to identify sepsis signs early. Efforts contributed to a 14% reduction in risk-adjusted mortality over a 2-year period.^17^

Establishment of PFACs has been recognized as a promising avenue to improve patient experience and safety, particularly in Singapore.^18^ SingHealth, the largest health care network in Singapore, partners with the SingHealth Patient Advocacy Network (SPAN)^19^—synonymous with a PFAC—to proactively engage patients in operational decisions, as patient advocacy and engagement are key elements of building an organizational culture of patient safety.^20^ Created in 2017, SPAN is a self-driven network of patients and caregivers that represents the collective voice of patients in care decisions. Members have participated in quality improvement, process design, patient experience, and facility design projects; curriculum development and delivery of training for health workers, residents and nurses; and speaking at SingHealth events as well as international conferences. SPAN also leveraged a survey, Care Partnership in Singapore, to identify gaps and opportunities to engage patients, caregivers, and health care professionals to implement solutions to promote patient safety.

SPAN Projects have included focus group discussions with patients and caregivers and reviews of leaflets provided to patients to ensure they are understandable and user friendly. For example, the network supported development of a Plain English Glossary^21^ of 150 commonly words and terms to make it more accessible and improve communication between patients and health care professionals. Improved communications between patients and clinicians have broadly demonstrated improved outcomes in the literature.^22^ SPAN efforts have also led to the creation of several PFACs in individual institutions within the SingHealth network and more continue to form at other hospitals and National Centers across Singapore.^23^

### Implementation considerations: bringing patients to the table and sharing insights

SPAN leaders recognized the importance of outreach to health care teams to communicate the need for patient engagement and make a case for how SPAN could support improvement efforts. To secure buy-in from the health care system, pilot projects were launched and subsequent learnings and best practices were shared.^23^ Patient advocates also attended quality improvement programs and participated in trainings to foster trust between clinical staff and patient advocates. SingHealth and SPAN have committed to ongoing measurement of the outcomes and impact of integrating patient and family engagement in patient safety and experience and plan to share insights on impact as they are identified. SPAN intends to use relevant indicators from the WHO Global Patient Safety Action Plan for 2021-2030, specifically from Strategic Objective 4: “Patient and Family Engagement,”^24^ to measure impact.^23^ Additionally, leaders can consider utilizing patient-reported experience metrics to assess impact. A number of OECD countries are co-developing surveys with patients to measure and monitor patient experience of safety incidents that empower patients and families to report to help prevent, evaluate, and manage patient safety incidents.^25^

Since its inception in 2017, SPAN has grown from a 13-member initiative to a current count of 50 patient and family advocates involved in over 100 projects.^23^ In an effort to support other health care organizations in developing patient engagement and advocacy capabilities, SPAN published the “Engagement Toolkit with Patients & Families for Healthcare Improvement Projects”^26^ to share insights into the work the network has done to engage patients and families to promote safety. Engaging patients and families in care decision-making promotes trust in providers to improve communication and provide a unique perspective to clinical decision-making catered to patients' unique needs. SPAN is a leader in the region and has shared the model across Singapore and with the World Health Organization Regional Office and is also considering the development of patient engagement modules to broadly disseminate learnings and engagement strategies.

## Leveraging technologies for improving patient safety

Emerging technologies can prevent or limit patient safety events while strengthening the capabilities of existing staff and empowering patients and families to utilize digital health tools.^15^ This can be implemented in a variety of areas, including medication safety, video monitoring to assess surgical performance, or use of AI-aided detection in radiology to assist clinicians in review to manage large quantities of scans to prevent late diagnosis that can lead to worse outcomes.^27^

Medication safety, including preventable prescription errors and adverse drug events, is one of the most common causes of patient harm, and an area where technology can reduce rates of harm.^1^ Medication safety can be challenging to improve as it involves many different health care professionals from initial prescription, verification, dispensing, and administration. All these lead to complexity and the potential for errors that impact patient safety. Traditional rule-based alerts used in current clinical decision support (CDS) systems, which aim to identify potential medication errors and safety concerns, have been found to cause alert fatigue and disrupt workflows.^28^ Additionally, CDS systems based on predetermined databases and rules have been reported to miss errors types that have not been pre-programed in the software rules, known as outliers.^29^ The use of machine learning decision support tools to support clinicians in medication safety decision-making and intervention efforts has increased in recent years.

### Evidence in action

Sheba Medical Center in Israel has implemented a medication safety initiative that leverages machine learning to minimize unnecessary medication safety alerts to reduce alert fatigue and improve clinical relevance of alerts. Sheba leverages the MedAware^30^ AI-enabled medication safety monitoring platform to identify preventable medication-related risks. The platform has been validated on more than 7 000 000 lives and has been shown to increase the number of relevant alerts generated. Sheba investigated the clinical effects of implementing MedAware and found it to have high accuracy, low alert burden, and low false-positive rates, leading to changes in subsequent orders.^31^ When compared with Sheba's legacy rule-based CDS system, the MedAware system generated close to 100 times fewer alerts that were 5 times more clinically relevant and caused over 8 times as many prescribing changes.

Another study involving 2 academic medical centers in Boston, Massachusetts, USA that utilized MedAware to identify potential errors generated a total of 10 668 alerts and found that 68.2% of MedAware alerts would not have been generated by the existing CDS system.^32^ Ninety-two percent of a random sample of the chart-reviewed alerts were accurate based on structured data available in the record, and 79.7% were clinically valid. The estimated cost of adverse events potentially prevented in an outpatient setting was more than $60 per drug alert and $1.3 million when extrapolating to the full patient population.

### Implementation considerations

Health systems and leaders interested in leveraging technology like machine learning used in the MedAware system to reduce errors and improve patient safety should consider technology implementation as important as the technology itself. This is especially important as human factor design and provider communications can determine adoption. Technology will also need to be implemented in conjunction with redesigned workflows that can minimize or even eliminate potential sources of human error. Furthermore, it will be important to ensure that health care providers are not over reliant on a new technology, recognizing that they continue to play a role in medication safety and to monitor for new patient safety issues (like cybersecurity).

Additionally, health systems continue to face external pressure to reduce costs while maintaining quality. Rising health care costs across the world create barriers to investments in technology and other resources for health care organizations grappling with cost concerns. Notably, the economic environment in countries across the world has become increasingly challenging,^33^ underscoring the challenges and tradeoffs organizations face in decision-making around technological investments in health care. There can be a perception that investing in technology requires upfront investment without guarantee that organizations share in savings. However, recent studies provide evidence that inpatient harm results in negative financial outcomes for hospitals (and payers), in addition to negative clinical outcomes for patients.^34^ As health care leaders consider the use of technology to address patient safety issues, key decision points will likely focus on the return on investment of such technology, including implementation costs. Evidence continues to be generated that demonstrates cost savings to health systems that offset the initial and ongoing investment in technology across various initiatives, including medication management.^35^ Additional insights will be gathered as the return on investment of use of AI or machine learning in hospitals continues to be assessed. Recent analyses have found AI use in clinical workflows can provide gains for hospitals through optimization of diagnostic and therapeutic procedures to improve care for patients. In the case of one hospital in the United States, AI use to support a radiology workflow resulted in reductions of 16 days of waiting time and 78 days of triage time.^36^

It will be important to continue to generate real-world evidence and ensure that new technologies achieve similar results in new systems, which may have different data systems with data stored in different formats. A key consideration, and potential challenge, when using machine learning alert systems is that performance is dependent on completeness and quality of underlying data screened in the system. When a health system faces collection challenges resulting in incomplete databases, the accuracy and validity of alerts may be impacted.^37^ Effective utilization of machine-learning technology to address medication errors will depend on data access and quality.

Another challenge is to ensure that new technology does not introduce or further exacerbate disparities in access or quality. This may occur because only well-resourced health care delivery organizations can afford the most innovative technologies or support access to high-quality and complete data, which may limit quality at less-resourced organizations. Additionally, disparities in access to technology can cause diagnostic inequities among patients due to barriers in timely diagnosis and referral.^38^ Furthermore, it will be important to monitor implicit biases that could lead to disparities in outcomes or safety. As innovative technologies continue to be implemented by health care organizations across settings, there are opportunities to address disparate access for under-resourced systems. Opportunities could include policies to incentivize utilization of technologies through provision of payments or resource supports to allow less-resourced organizations to develop required infrastructure. Development of such incentives may require partnerships with policymakers.

## Scaling strategies across settings to advance patient safety

Scaling strategies and innovations to advance patient safety across individual organizations, as well as in different countries across the world, will involve collaborative efforts among stakeholders, including health systems and policymakers. At an organizational level, it is important for leaders to acknowledge hospitals and health systems have different cultures, management, and expectations related to patient safety interventions. For example, safety efforts may be led at the unit management level, by quality safety teams, or at the regional or system level. It is imperative to meet patient safety teams where they are to support sustainable implementation of new decision support tools, facilitate patient and family engagement, and implement new technologies.

Successful scaling of strategies to advance patient safety at the macro or country-level will need to account for individual country characteristics, particularly as resources and capabilities vary across countries. Health system and policy leaders can work together to collect and synthesize findings from implementation of emerging and innovative technologies to identify impact and opportunities to scale efforts across settings. Strategies and innovations that prove effective in improving outcomes and reducing costs may generate support from payers and providers. Health care organizations can consider partnerships with government entities to support wide-spread adoption of innovative strategies or coverage of technologies to address cost-related barriers and, ultimately advance patient safety.

## Conclusion

Although health systems around the world have made significant strides in improving patient safety and minimizing preventable harm, much more is needed. This paper identifies specific case examples and implementation strategies that can be used now by health care delivery systems, technology companies, patient groups, professional associations, and others to reduce the stubbornly persistent rates of patient harm. This paper also highlights the multi-faceted nature of patient safety, which requires better measuring, in real time, the rates of patient safety; engaging patients and families in addressing patient safety; and better leveraging technologies to reduce potential errors. Looking to the future, there will be more effective strategies for improving patient safety, and additional opportunities to build on the learnings to date for health care leaders to strive to avoid preventable patient harm and improve outcomes.

## Supplementary Material

qxag008_Supplementary_Data
